# CATCH-EVseq, an Ultra-Sensitive Capture Assay to Monitor Human Enterovirus RNA

**DOI:** 10.3390/ijms27209065

**Published:** 2026-10-13

**Authors:** Arnaud Germain, Maureen R. Hanson

**Affiliations:** Department of Molecular Biology and Genetics, Cornell University, Ithaca, NY 14853, USA; ag297@cornell.edu

**Keywords:** enterovirus (EV), virus detection, myalgic encephalomyelitis, ME, ME/CFS, RNAseq, chronic infection

## Abstract

The detection of low-abundance viral RNA in chronic or persistent infections remains a major challenge in virology, constrained by the sensitivity and specificity of traditional methods. One such infection, chronic enterovirus (EV) infection, has been proposed as a potential underlying factor in Myalgic Encephalomyelitis (ME), although this hypothesis remains debated. Supporting evidence for EVs as a viral trigger includes historical spatiotemporal overlaps between ME outbreaks and poliomyelitis epidemics, frequent reports of “flu-like” illness at disease onset, seasonal outbreaks patterns, and serological findings. To address the problem of EV detection, we developed CATCH-EVseq, an ultra-sensitive platform built on the CATCH framework. By integrating high-throughput sequencing with cDNA capture using 49,847 probes targeting 3410 EV genomes, CATCH-EVseq achieved a detection limit in artificially created samples as low as one copy in a microgram of RNA for two out of three biological replicates. Additionally, spike-in controls allow for rigorous validation of the capture and sequencing throughout the CATCH-EVseq procedure. When applied to a small number of blood samples from both ME patients and healthy controls, CATCH-EVseq detected no EV RNA, either in direct assays or after in vitro viral amplification. While these results do not support the presence of circulating EV RNA in the blood of individuals with ME, they do not rule out persistent infection in other tissues or organs. Critically, CATCH-EVseq establishes a robust, ultra-sensitive framework for future investigations of low-level EV persistence in biopsies, post-mortem tissues, or other clinical contexts where chronic enterovirus infection is suspected.

## 1. Introduction

A longstanding obstacle in virology has been the detection of low-abundance viral RNA in chronic or persistent infections, where the sensitivity and specificity of traditional methods fall short. Enteroviruses (EVs), among the most prevalent viral pathogens globally, are responsible for an estimated 10–15 million non-polio infections annually in the United States alone, according to the Centers for Disease Control and Prevention [1]. They have been implicated in a range of acute and chronic diseases, including viral cardiomyopathy, type 1 diabetes, post-polio syndrome, and acute flaccid myelitis [2,3,4,5]. Recent outbreaks of serotypes such as Coxsackievirus A16, A6, and A24, EVA71, EV70, echovirus 13, 18, and 30, and EVD68, linked to conditions like ‘hand, foot, and mouth disease’, conjunctivitis, meningitis, acute flaccid myelitis, and severe respiratory illness, have further highlighted their capacity for persistence and chronicity [1]. Disease outcome is shaped by host immune susceptibility [6,7], EV serotype and quasispecies diversity [8], tissue and cellular tropism, and viral adaptations that support persistence.

Historical observations, including the spatiotemporal overlap of ME outbreaks with poliomyelitis epidemics, the frequent reporting of “flu-like” illness at disease onset, and the seasonality of cases, have long suggested a viral origin, as summarized in comprehensive reviews [9,10,11]. Symptoms associated with EV infections, particularly those affecting muscle and nervous system tissues, substantially overlap with ME, raising the possibility of ultra-low-level or tissue-restricted EV involvement. However, while many studies employing serological, immunochemical, tissue culture, and molecular approaches have reported elevated EV markers in ME cohorts, methodological inconsistencies and variability in assay sensitivity have complicated definitive conclusions [9]. Most prior investigations have focused on tissue-based analyses (e.g., muscle, gastrointestinal, and central nervous system specimens), with relatively few systematically evaluating circulating EV RNA in peripheral blood using high-sensitivity techniques. Furthermore, even when RT-PCR methods detect enterovirus, the viral sequence information is quite limited because of the necessity to use primers corresponding to conserved regions.

To address these challenges, we employed target capture enrichment to enable comprehensive detection of known and potential undiscovered EVs at sensitivity exceeding conventional RNA sequencing [12]. Using the CATCH framework (Compact Aggregation of Targets for Comprehensive Hybridization) established by Metsky and his colleagues [13], we developed an ultra-sensitive platform named CATCH-EVseq. By integrating high-throughput sequencing with cDNA capture, using 49,847 probes targeting 3410 EV genomes from EV species A–D, CATCH-EVseq achieves unprecedented sensitivity, validated through rigorous viral spike-in controls.

While CATCH-EVseq did not detect EV RNA in the blood from the small number of ME patients we analyzed, this does not rule out EV involvement in the disease, as persistent infection may be restricted to other tissues [14]. Defining these sensitivity limits is essential for refining models of EV persistence and guiding future investigations into tissue reservoirs and post-mortem specimens, ultimately advancing our understanding of ME pathogenesis.

## 2. Results

### 2.1. Probe Design and Localization

**Figure 1:**
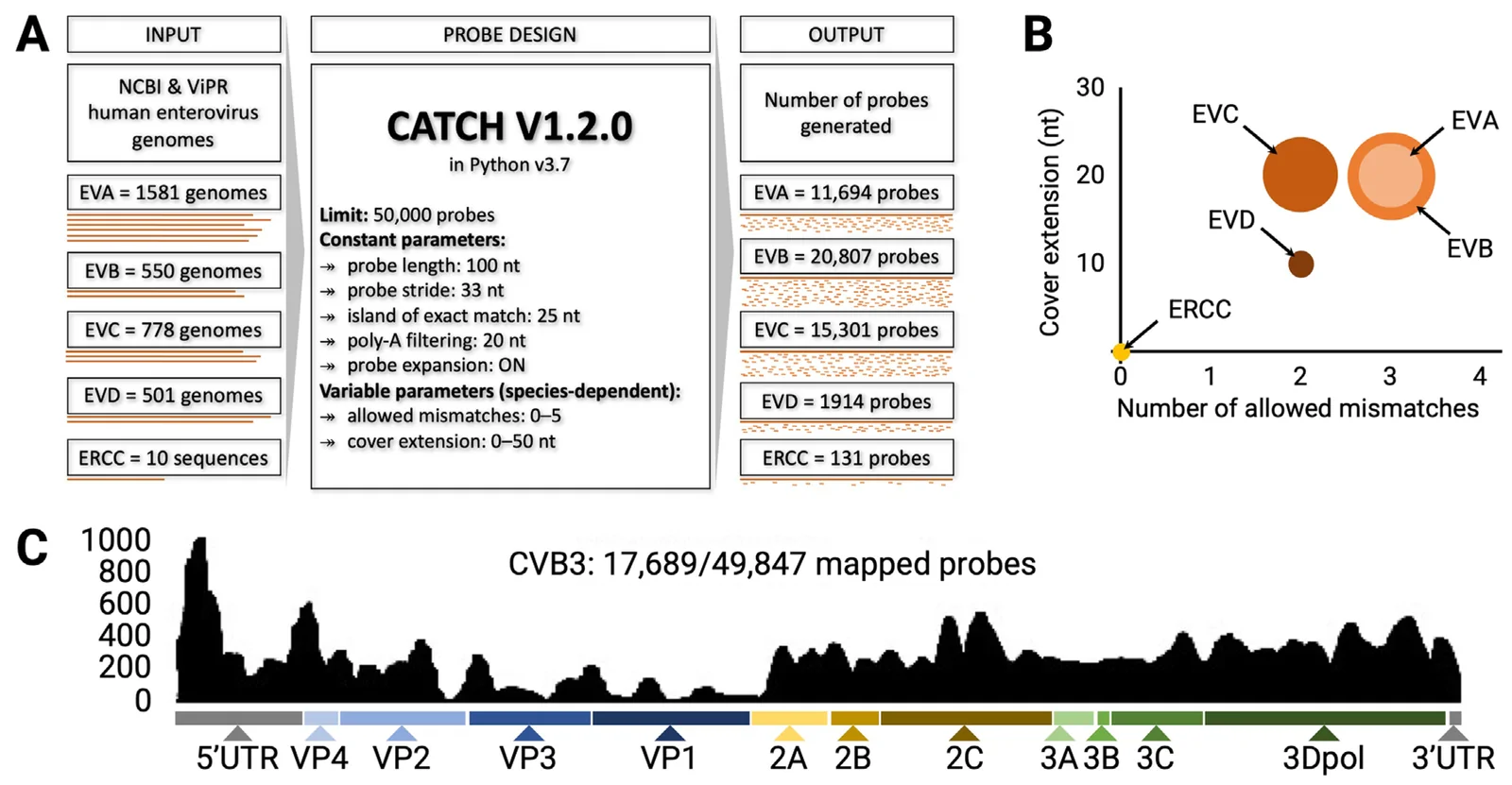
Design, parameters, and genomic localization of EV-specific probes using the CATCH framework. (**A**) CATCH-probes workflow design, including input, parameter and output numbers. (**B**) Bubble plot of species-specific variable parameters in CATCH-probe design for EVA–D and ERCC controls. Bubble size reflects the number of probes generated for each group. (**C**) Genomic distribution of CATCH-probes designed across CVB3. The *y*-axis represents number of probes (coverage depth).

CATCH’s framework allows users to input a database of their choice for the algorithm to use to design probes with hybridization characteristics that will allow samples with the diversity of the chosen sequences. The input comprised 3410 EV genomes, including 1581 EVA, 550 EVB, 778 EVC and 501 EVD species, along with 10 ERCC (External RNA Controls Consortium) spike-in sequences. A total of 49,847 probes were devised using the CATCH framework (Figure 1A) [13]. The complete list of EV genome sequences and lengths, as well as ERCC details, along with probe sequences, are provided in Supplementary Table S1. The CATCH framework also allows selection of the total number of probes to be designed, since financial constraints and database diversity will affect what is both feasible and required. We selected the number of probes to be 50,000, with a probe length of 100 nucleotides with up to five mismatches permitted (Figure 1A).

CATCH will generate probes that differ depending on the characteristics of the sequences to be captured. In addition, CATCH will take into account the cover extension, which is an indication of how much of the target DNA on each side of the hybridized region will be expected to be included in the captured fragment. The four EV families differ in the number of known sequences, as well as in their sequence variability. For EVA and EVB, our specified design allowed three mismatches with a 20-nucleotide cover extension, while EVC probes were designed with two mismatches and a 20-nucleotide cover extension. EVD probes permitted two mismatches with a 10-nucleotide cover extension, and ERCC-directed probes had zero mismatches and no cover extension (Figure 1B).

In silico mapping of the CATCH-designed probes demonstrated variability in mapping density across genomes. This variability in coverage depth stems from the algorithmic optimization within CATCH, particularly the application of the “set cover problem”, which limits the number of probes while prioritizing regions of high genomic variability. Consequently, fewer probes were generated for conserved regions. As an illustrative example, 17,689 of the 49,847 probes mapped to CVB3 with the majority localizing to the 5′UTR, P2 (2A–2C) and P3 (3A–3D) genomic regions (Figure 1C). This distribution is justified, as these regions are recognized as recombination hotspots across EV species [15].

### 2.2. CATCH-EVseq Workflow

**Figure 2:**
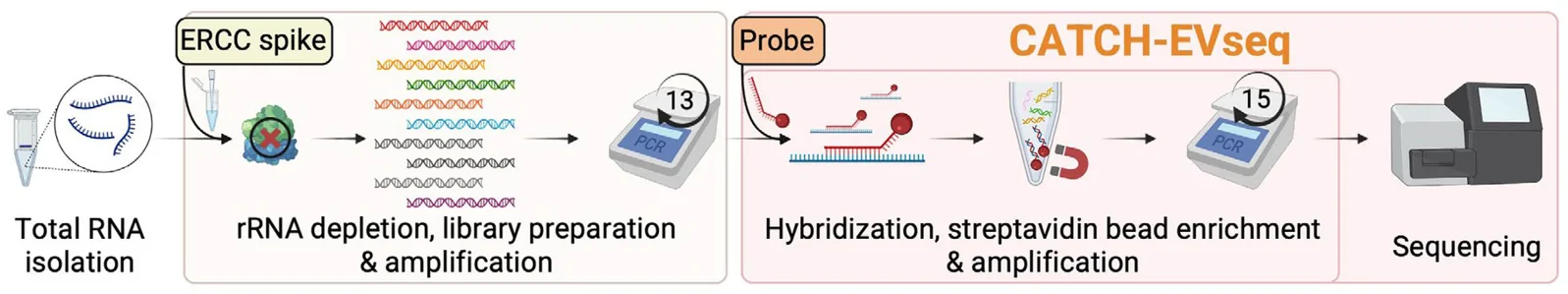
CATCH-EVseq workflow generated using biorender.com. The Illumina library preparation and sequencing include ERCC RNA spike-ins and rRNA depletion, followed by EV sequences enrichment. Additionally, two rounds of amplification are performed: 13 cycles during library preparation and 15 cycles during CATCH-EVseq.

Our workflow begins with the isolation of total RNA, followed by ERCC RNA spike-in and ribosomal RNA depletion during the library preparation, and a PCR amplification step (13 cycles, Figure 2). The enrichment of EV sequences using our targeted hybridization probes and streptavidin beads was performed on pooled cDNA libraries (maximum of eight), according to the manufacturer’s recommendation [16]. Post-enriched libraries were sequenced following an additional PCR amplification step using Illumina primers (15 cycles).

Downstream analysis of the paired-end read datasets first included filtering out known non-target reads (e.g., human or cell culture). Secondly, the assay’s performance was validated based on the recovery rate of the ERCC transcripts. The remaining sequences were then aligned against our database of 3410 human EV reference genomes (Supplementary Table S1), enabling precise identification and visualization of viral sequences.

### 2.3. Assay Sensitivity

To establish the lower limit of detection (LOD) of our assay, we spiked human heart total RNA with a 1:10 serial dilution series of EV71 RNA, ranging from 1000 down to one viral copy per microgram of background RNA. Sequencing on a NovaSeq600 sequencer (illumina, Inc., San Diego, CA, USA), with a target of 10 million reads, generated 2 × 150 base paired-end reads. This setup allowed us to evaluate, in biological triplicates, the sensitivity and robustness of EV71 genome recovery across decreasing viral counts.

**Figure 3:**
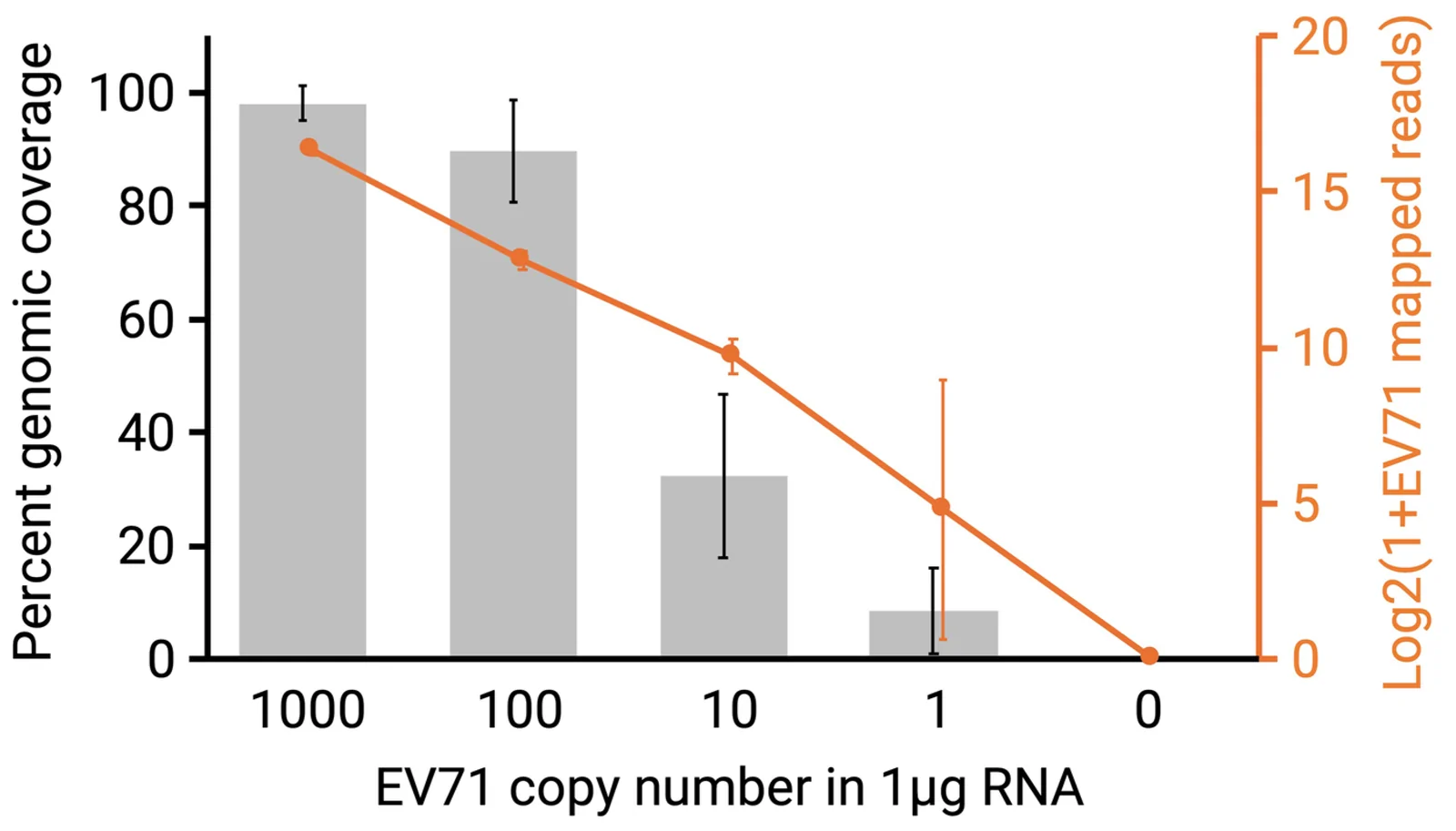
Relationship between EV71 dilution concentration and mapped reads recovery. Graph displays the mean percent coverage of the EV71 genomic sequence (grey bars and their mean ± standard deviation), as well as the mean number of recovered mapped reads (orange line) as a function of input concentrations conducted in biological triplicates. The *x*-axis indicates the EV71 concentration in copies per microgram of total RNA, with “0” representing the negative control.

At the highest input of 1000 EV71 copies per microgram of RNA, we achieved near-complete genome recovery (Figure 3), with an average coverage of 98.5 ± 2.3% (mean ± standard deviation), while 100 copies had an average coverage of 89.8 ± 9.2%. This level of recovery demonstrates the assay’s effectiveness at detecting and almost fully recovering the complete genome sequence of EV71 when present in sufficiently abundant concentrations.

**Figure 4:**
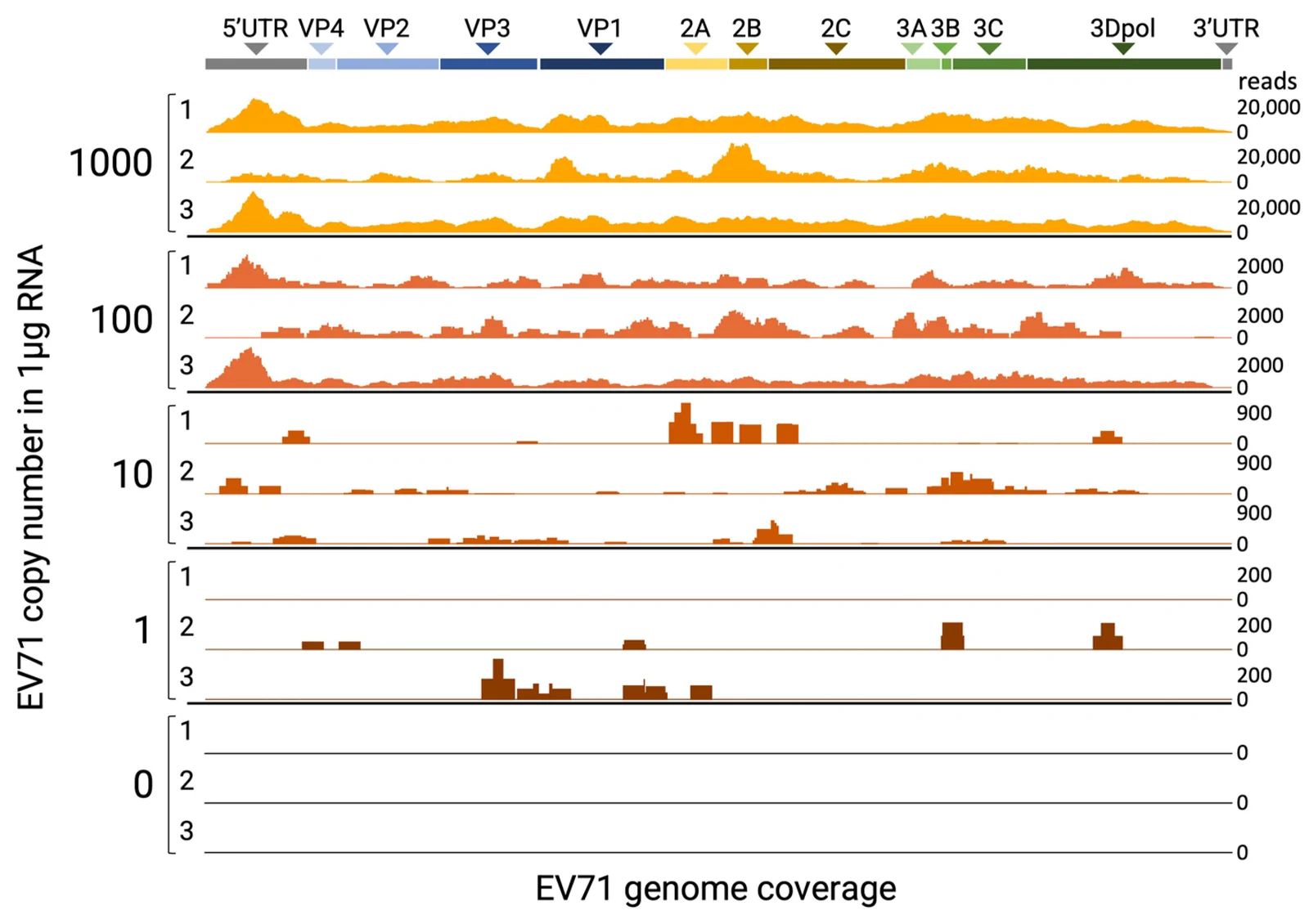
Visualization of the read coverage of the EV71 genome for the EV71 dilution series. IGV was used to display the regions of the genome covered by reads for each of the biological triplicate sequencing runs, with read-counts on the *y*-axis to the right of the bar-plots.

Another way to visualize the recovery rate of the EV71 genome is to map the read-counts to the EV71 reference genome. The results are displayed in Figure 4 and confirm the high level of recovery for the 1000 and 100 EV71 copies biological triplicates. For the 10 EV71 copies per microgram of RNA, the average genomic coverage fell to 36.5 ± 15.7% (Figure 3), with more sporadic coverage and lower read-counts (Figure 4). At the lowest input level of one EV71 copy per microgram of RNA, detection was achieved in two of the three samples, with recovered genome coverages of 11.2% and 14.6%, and five sequenced locations for triplicate #2 and #3 (Figure 3 and Figure 4). Combined, these results highlight the assay’s LOD, with the capacity to detect EV71 even at minimal concentrations, albeit with increased variability at the single-copy level.

### 2.4. Screening for EVs in Whole Blood

**Figure 5:**
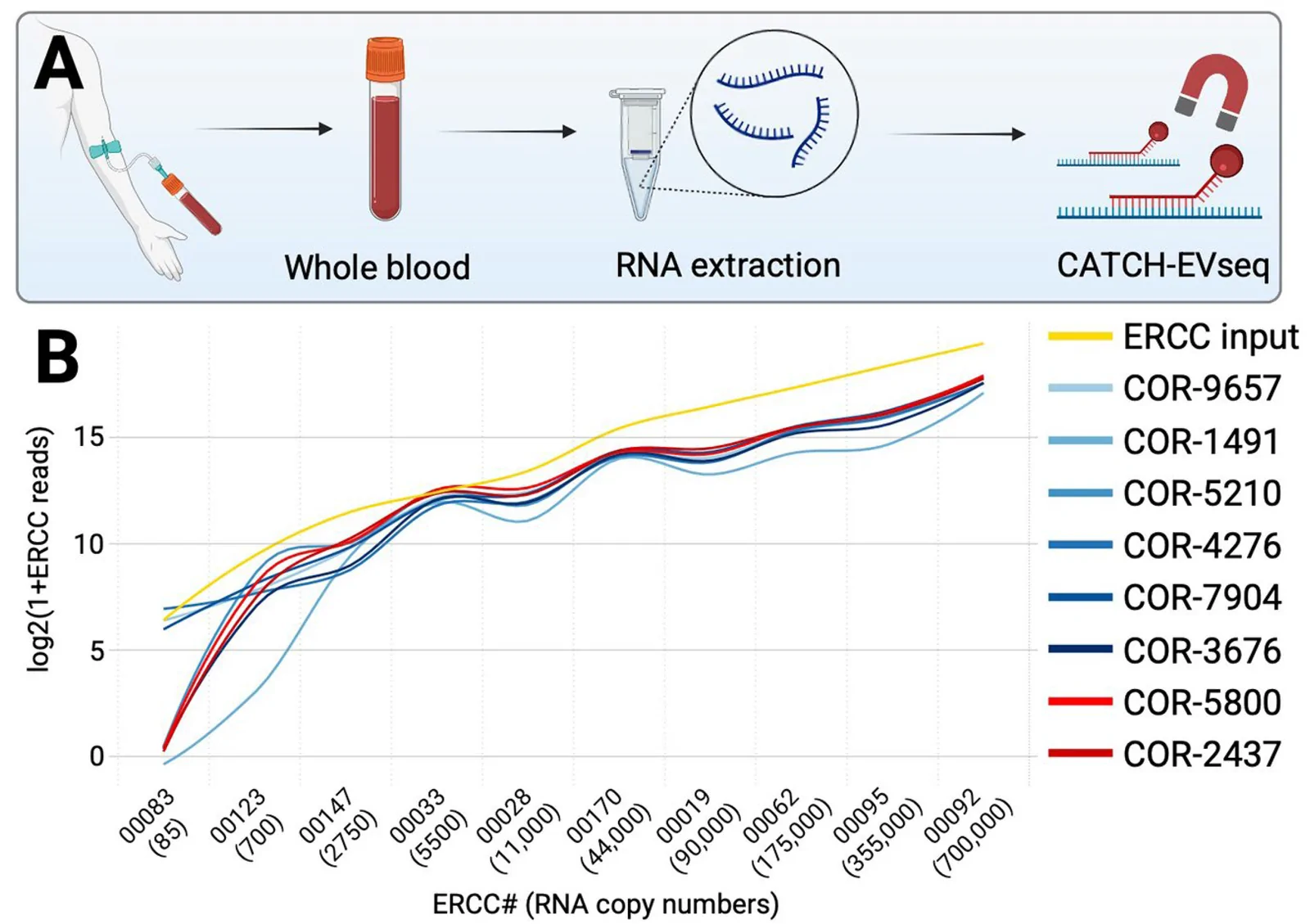
CATCH-EVseq on whole blood. (**A**) Workflow for whole blood EV hunting generated using biorender.com (**B**) Graph displays the recovery rate of ERCC transcripts in a 250 ng RNA background at a target depth of ~2 million reads per sample. ME samples are depicted in blue and controls in red.

Our initial EV screening experiment was performed on whole blood RNA from six ME subjects and two controls (Figure 5A), using 250 ng of RNA at a target sequencing depth of ~2 million 2 × 250 base paired-end reads (MiSeq, illumina, Inc., San Diego, CA, USA). Using whole blood allowed for the screening of both the plasma and cellular parts of the blood (red blood and immune cells).

**Figure A1:**
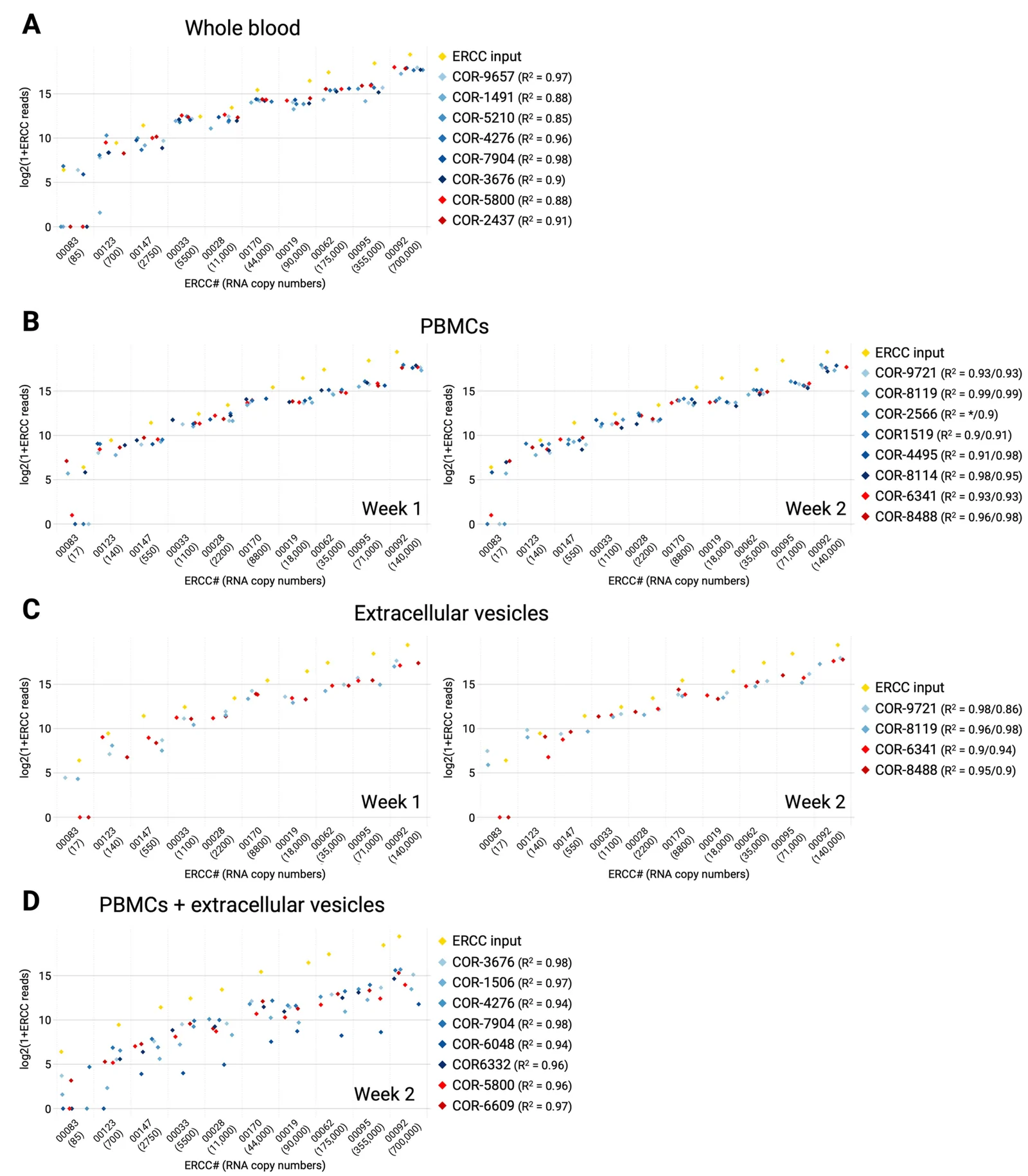
Dot-plot graphs of the recovery rate of ERCC transcripts in Figure 5B and Figure 6B–D. The R^2^ of the linearity assesment between read counts and ERCC spike-in input concentration is reported next to each sample’s name. (**A**) Whole blood from Figure 5B. (**B**) Samples inoculated by PBMCs and harvested at both week 1 and week 2 from Figure 6B. The asterisk (*) denotes the absence of data for sample COR-2566 at Week 1. (**C**) Samples inoculated by extracellular vesicles and harvested at both week 1 and week 2 from Figure 6C. (**D**) Sample inoculated by subject-matched PBMCs and extracellular vesicles, harvested at week 2 from Figure 6D.

While no evidence of EV presence was detected, as part of our workflow (Figure 5A), we could further evaluate the efficacy of CATCH-EVseq using the recovery rate of spiked-in ERCC transcripts and compare it to the ERCC input. Nine of the ten probe-targeted ERCC transcripts were identified in all eight samples, albeit with only two ERCC00123 read counts in sample COR-1491 compared to the 700 copy-input (Figure 5B). For the least abundant transcript, ERCC00083 and its 85 copies, recovery detection occurred for three of our eight samples. Exact values are provided in Figure A1 (dot-plot format), as well as copy-counts in Supplementary Table S2.

Slightly inferior recovery rates compared to input occur for the ERCCs with the highest copy numbers (Figure 5B), potentially showing probe hybridization saturation.

Overall, these results indicate that reliable ERCC recovery occurs between 85 and 700 transcripts per 250 ng of RNA when sequencing at a depth of ~2 million reads per sample. Nevertheless, while ERCC RNA spike-in transcripts can serve as a valuable internal standard for assessing assay performance, their utility in quantifying EV transcript recovery is semi-quantitative due to their inherent size variability. Indeed, each ERCC transcript varies in nucleotide length (ranging from 521 to 2022 nucleotides, Supplementary Table S1), which naturally influences the number of dedicated sequencing reads per transcript. Furthermore, the mean length of EV genomes is 7329 nucleotides (median of 7394), while ERCC transcripts are considerably smaller, with the largest transcript, ERCC00033, being 3.7 times smaller. Comparing the ERCC recovery trends to the EV71 dilution series (Figure 3 and Figure 4) highlights such discrepancy, with 100% detection in 10 EV71-copy triplicates and 67% in one EV71-copy triplicates compared to a detection rate of only 37.5% for ERCC00083.

### 2.5. Screening for EVs Following an Attempt at Virus Amplification

**Figure 6:**
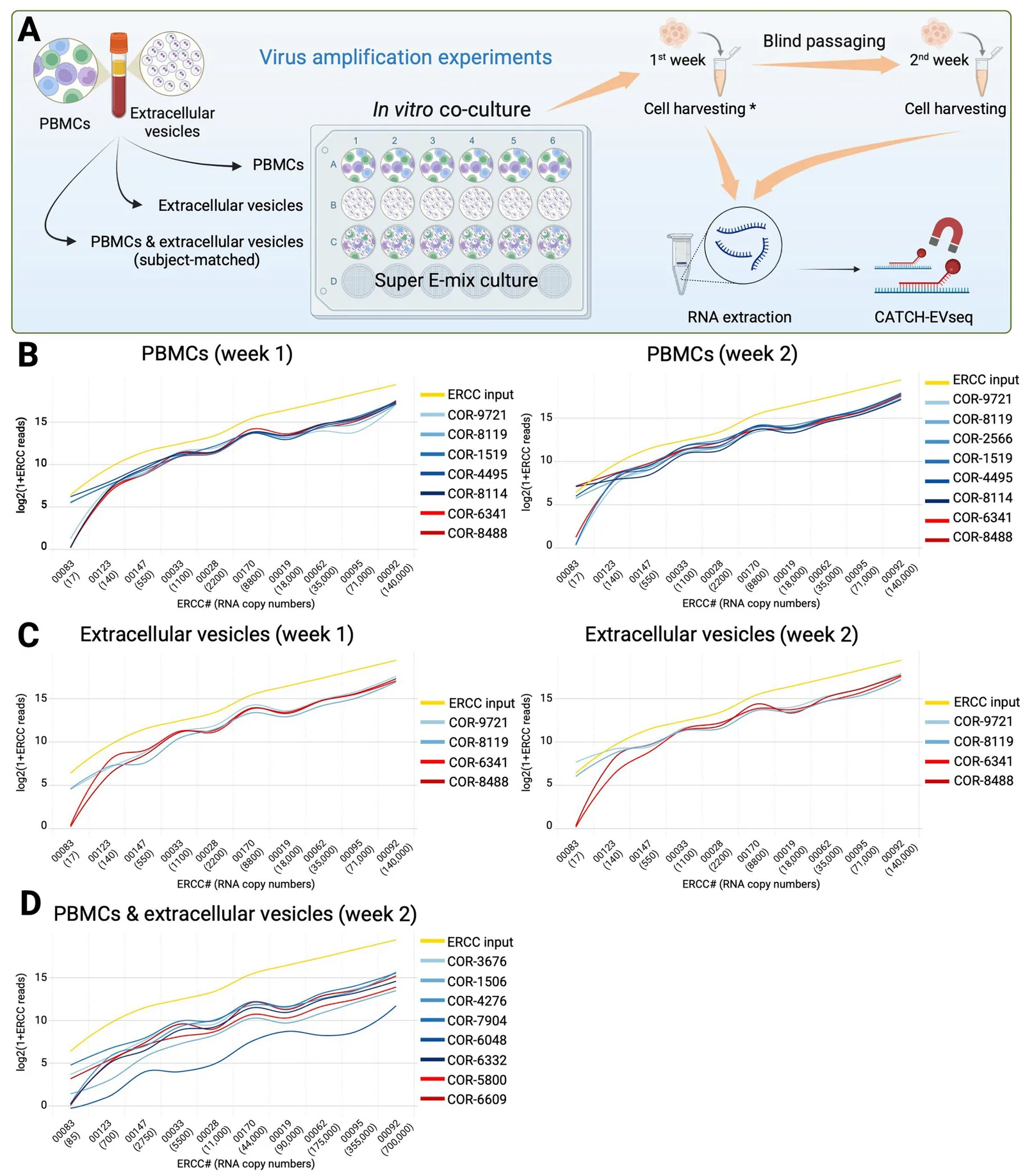
CATCH-EVseq following the virus amplification attempt. (**A**) Workflow of virus amplification generated using biorender.com. The asterisk (*) denotes that no cell harvesting was peformed after one week for the PBMCs & extracellular vesicles condition. (**B**) Graph displaying the recovery rate of ERCC transcripts in a one microgram RNA background at a depth of ~20 million reads in a sample inoculated with PBMCs and harvested at both week 1 and week 2. ME samples are depicted in blue and controls in red. (**C**) Graph displaying the recovery rate of ERCC transcripts in a one microgram RNA background at a depth of ~20 million reads in a sample inoculated with extracellular vesicles and harvested at both week 1 and week 2. ME samples are depicted in blue and controls in red. (**D**) Graph displaying the recovery rate of ERCC transcripts in a 500 ng RNA background at a depth of ~2 million reads in a sample inoculated with subject-matched PBMCs and extracellular vesicles and harvested at week 2. ME samples are depicted in blue and controls in red.

We attempted to cause amplification of EVs by incubating blood components with SUPER E-MIX^TM^ cell cultures. We inoculated cultures with peripheral blood mononuclear cells (PBMCs) from six ME subjects and two controls or extracellular vesicles purified from plasma from the same controls and two of the six ME subjects (Figure 6A). Cells were harvested after one week, and again after another week following blind passaging. One microgram of extracted RNA from cell pellets was sequenced (HiSeq, illumina, Inc., San Diego, CA, USA) at a target depth of ~20 million 2 × 75 base paired-end reads (Figure 6A). Independently, subject-matched PBMCs combined with extracellular vesicles from eight different subjects, six ME patients and two controls were also inoculated into SUPER E-MIX^TM^ cultures. Cells were harvested after two weeks of culture, including blind passaging after the first week (Figure 6A), before sequencing 500 ng of RNA on a MiSeq at a target sequencing depth of ~2 million 2 × 250 base paired-end reads.

**Figure A2:**
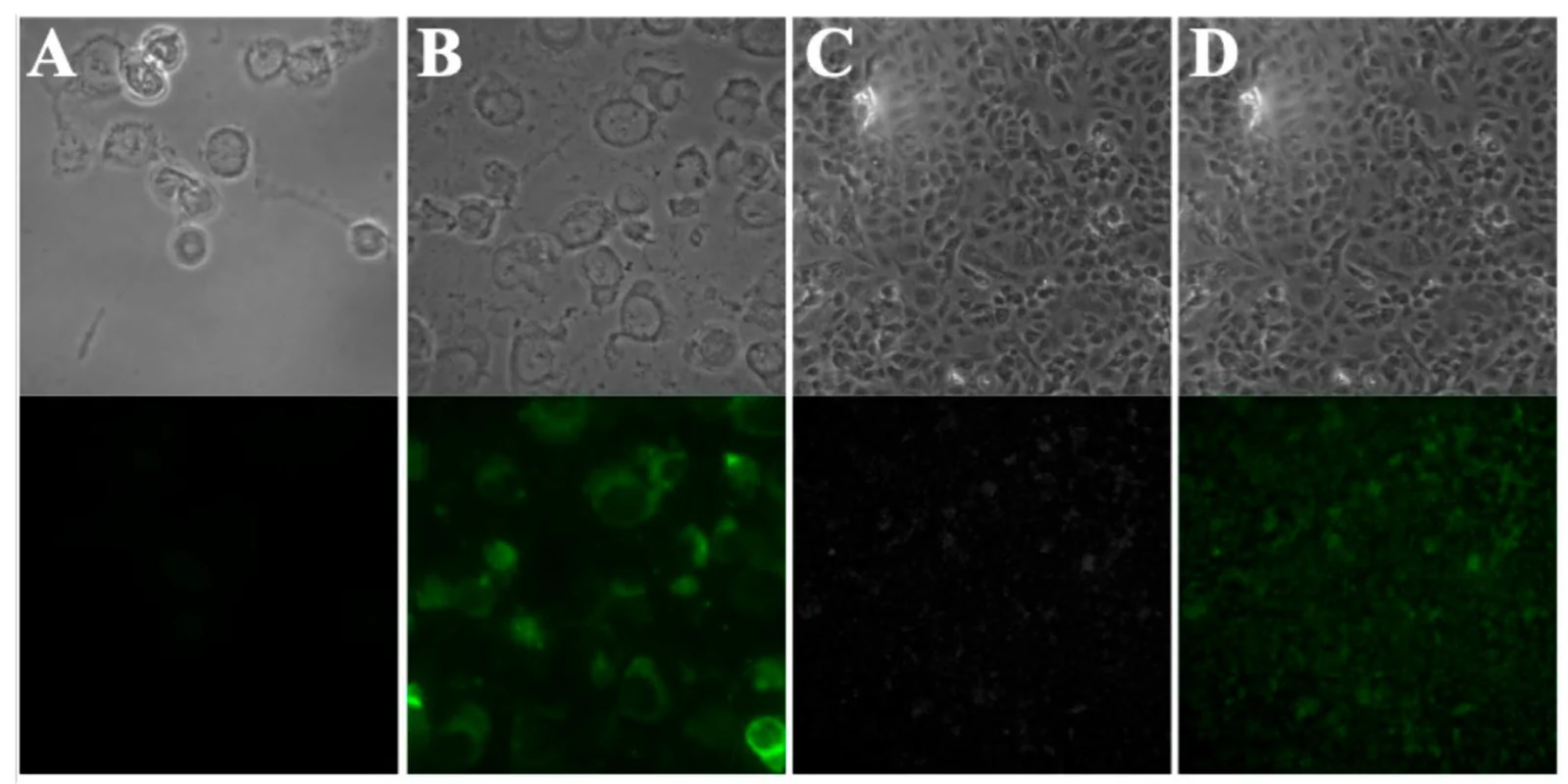
EV VP1 immunochemistry using the Quidel D^3^ IFA Enterovirus Detection kit (**Top**). Phase contrast images (**Bottom**). Immunofluorescent images. Green, punctate fluorescent signal indicates antibody reaction with VP1 in infected cell cultures. (**A**) Negative control staining at 1000× using EV antigen control slides. (**B**) Positive control staining of Coxsackievirus B infected cultures at 1000× using EV antigen control slides. (**C**) Representative image showing negative staining in cultures inoculated with case and control samples at 100× magnification. (**D**) Same image as C but with oversaturation to show a lack of punctate signaling even after excitation is raised to levels higher than control slide imaging.

Here, again, no EV presence was detected through sequencing at any timepoint across all experiments. Daily monitoring of cell cultures for signs of EV infection revealed no visible cytopathic effect (CPE). Immunocytochemistry (ICC) performed at the times of cell harvest, did not uncover the presence of the EV VP1 protein (Figure A2).

We could, however, use those five supplementary datasets to further assess instances of ERCC spike-in recovery rates compared to known ERCC RNA inputs (Figure 6B–D). The trends observed for the PBMCs (Figure 6B) and extracellular vesicles cell culture experiments (Figure 6C) at both timepoints were very similar to the whole blood EV screening data (Figure 5B), with reliable ERCC recovery of nine out of the ten probe-targeted ERCC transcripts in all samples. The recovery of ERCC00083 (the least abundant ERCC transcript) was successful for 13 out of the 23 samples (57%), albeit with only one read count in samples COR-9721 and COR-6341 for PBMCs cell culture inoculations at week 1 and week 2, respectively (Figure 6B), compared to the 17-copy-input per microgram of background RNA. Regarding the PBMCs and extracellular vesicle co-inoculation, even though the trend is similar to all the other datasets, the enrichment seems to have been less efficient and is most likely due to human error, as the same reagents and procedures were used. Exact values are provided in Figure A1 (dot-plot format), as well as copy-counts in Supplementary Table S2.

These results highlight the robustness of ERCC spike-ins as internal controls for assessing assay performance, while also emphasizing the challenges associated with detecting low-abundance transcripts in complex biological samples.

### 2.6. Exploration of the Sequence Data

Our analysis employed a multi-layered, redundant approach to ensure no potential EV reads were overlooked. We began with a state-of-the-art workflow: standard QC and trimming (FASTP), followed by ERCC transcript removal (bowtie2) and host genome depletion (human or buffalo green monkey kidney cell line reference, using STAR). The remaining reads were then aligned against our database of 3410 human EV reference genomes (bowtie2). The ERCC read counts from the first two steps are presented in Figure 5B and Figure 6B, Figure A1 and Supplementary Table S2.

To further validate our results, we executed an alternative workflow, directly aligning raw reads against the EV genome database. This unusual approach also yielded no EV read hits.

Finally, we leveraged CZID (czid.org), an open-source platform for infectious disease surveillance, to analyze our sequencing datasets. This step aimed to expand our EV reference genomes and screen for other known viral pathogens. While no viral reads were detected, a few incidental bacterial reads were identified, with no distinction between ME patients and controls.

## 3. Discussion

We designed CATCH-EVseq, a capture-based enrichment method enabling the comprehensive detection of known EVs, as well as potentially undiscovered EVs, at a sensitivity exceeding conventional RNA sequencing. In total, 49,716 probes are used to grab EV sequences from Illumina libraries and further amplify them before sequencing. These probes were devised using the platform CATCH [13], using all known human EVs labeled as complete available from the NCBI and ViPR databases (Figure 1A). An additional 131 probes target a subset of 10 ERCC transcripts spiked in the RNA before library preparation and are used as internal standard to ensure assay performance if no EV reads are detected.

We demonstrated the efficacy of CATCH-EVseq by hunting for EV71 in one microgram of EV71 spiked-in RNA (Figure 3 and Figure 4). Recovery was successful in two out of three samples with one copy of EV71, at genome coverages of 11.2% and 14.6%, which is enough to confirm the presence of EV, even if not enough to recover the full sequence. Considering most commercial kits estimate yields of one to five microgram of total RNA from one milliliter of whole blood in PAXgene tubes [17], we can entertain some estimates for the LOD in a clinical context. Consequently, CATCH-EVseq can detect as low as one EV copy in one milliliter of blood, with 67% confidence. We can extrapolate to 100% confidence at slightly higher EV copies, potentially two to 10 EV copies in one milliliter of blood, since we obtained coverage of multiple genome regions in 10 EV71 copies samples (Figure 3 and Figure 4).

The sensitivity of CATCH-EVseq, an EV-specific set of probes, can be compared to the sensitivity of the 349,998 probes (V_ALL_) targeting 356 species of viruses with reported cases of human infections, that were generated by the creators of CATCH [13]. The authors did an identical serial dilution using the Ebola virus and their V_ALL_ probe set and found reliable detection of viral content down to 100 copies in a 30 ng of background amount of human RNA at a depth of 200,000 reads, which is one order of magnitude lower than without capture. While this sensitivity is lower than in our case, the authors note that higher sequencing depths, like ours at two million reads, would have provided more viral material and thus increased the sensitivity in detection. Interestingly, they report that a two-virus (MuV and ZIKV) probe set V_MM_ and V_ZC_ enriched unique viral reads 2.26 times more than V_ALL_. While this gain was dismissed as useful only in some applications, based on their purpose of a widely viral inclusive V_ALL_, they further evaluated the capture efficiency of sequences divergent from the sequences used in the probe design. They ultimately report that sequences markedly differing from their designed targets were also captured. The high sensitivity of CATCH-EVseq could therefore be attributed to higher sequencing depth, as well as a high number of probes dedicated to one viral species. In the case of V_ALL_, roughly half the number of probes (24,728) are dedicated to human enteroviral targets, requiring an increased cover extension and greater number of allowed mismatches [13].

An earlier but equivalent target capture technology to V_ALL_, VirCapSeqVERT, targeted 207 viral taxa known to infect humans with an alternative approach to probe design, although the probes sequences were not released to the public [18]. They report that VirCapSeqVERT can detect 10 EVD68 copies in a milliliter of blood with an average of 34 out of 10 million reads at an average genomic coverage of 8.2%. CATCH-EVseq is therefore up to 10 times more sensitive at a sequencing depth five times lower, for a comparably sized EV target, EV71 (Figure 3 and Figure 4).

The strength of both VirCapSeqVERT and CATCH-EVseq is in identification of unknown or highly mutated viruses for which specific probes have not yet been designed. Neither are appropriate for use for routine diagnosis of known viruses, since less expensive and technically less demanding methods such as RT-PCR and qPCR could be used instead.

While we did not detect EV genomic RNA in the blood of ME cases both before and after in vitro attempts of viral amplification using CATCH-EVseq, a lack of EV detection does not prove a lack of chronic enteroviral infection. In the case of pathologies associated with chronic EV infections, the viruses are known to reside in a persistent state within tissue sites such as the central nervous system, muscle and pancreas [2,3,4,5]. Many chronic enteroviral infections caused by myotropic and neurotropic EVs invoke a symptom constellation profile consistent with ME [9]. The World Health Organization (WHO) guidelines on non-polio EV surveillance directs researchers to more appropriate preferred and alternative sample types based on clinical syndrome presentations [19]. In the case of ME, the WHO suggests EV surveillance should initially be focused on brain tissue and cerebrospinal fluid and secondarily to feces, throat swab, oropharyngeal swab, nasopharyngeal swab, and rectal swab [19]. To settle the question of chronic EV infection in ME, we need to use CATCH-EVseq, an integrated, comprehensive approach for sensitive EV detection, on sample types such as brain, cerebrospinal fluid, muscle, and gastrointestinal tissues.

Moreover, we want to suggest to future users a potentially modest but obvious enhancement relating to the ERCC spike-ins and the probes targeting them. Indeed, now that we thoroughly validated CATCH-EVseq, it seems superfluous to keep enriching for high-copy numbers of ERCCs in future experiments, which ultimately pollute the sequencing files used to look for EV reads. Retaining the probes for the least abundant ERCCs, perhaps the lower four or five, would retain experimental validation while theoretically increasing the sensitivity of CATCH-EVseq at similar sequencing depths, or allow for the reduction in sequencing costs by lowering the sequencing depth.

The detection of EV sequences has been positive using RT-PCR in four studies using blood, six studies using muscles, two using brain tissues, and one study each using throat swabs, stomach tissues or heart tissues [9]. The four studies detecting EVs in blood were, however, performed with samples collected in the early 1990s, not long after major world-wide outbreaks of ME [9,10,11], unlike most samples collected pre-2020 today, which usually come from individuals with long illness duration. The WHO recommends the use of the PanEV primer pair to screen for EV infections from nearly all types of EVs [20]. Those primers target a highly conserved region in the 5′ end of the EV genome and would therefore rely on the presence of this region (no mutation or deletion) and a higher detection threshold compared to CATCH-EVseq. Given the greater likelihood of detecting persistent enterovirus in tissue, a more cost-effective method for assessing persistent infection could be to perform sample pre-screening using the more economical RT-PCR method, then follow analysis of positive samples with CATCH-EVseq to outline full EV sequences, their mutations, and enable the simultaneous characterization of EV co-infections. We hope to obtain tissue samples in the future for further studies of EV persistence in ME.

In summary, CATCH-EVseq proves to be acutely sensitive, with the added benefit of being comprehensive in nature, allowing for the survey of both known and potentially novel human EV sequences at a sensitivity yet to be employed in the field of ME. Incidentally, a recent manuscript regrouping many recommendations from a group of experts as part of the European non-polio enterovirus network (ENPEN) proposes, amongst many options, a similar approach used here when designing the full CATCH-EVseq workflow [21]. CATCH-EVseq, combined with the use of EV-susceptible cultures to perform in vitro viral amplification, is a promising tactic to employ in future ME EV-related studies [22].

## 4. Materials and Methods

### 4.1. Cohort Demographics

This cross-sectional study involved five controls and 15 ME subjects diagnosed using the Canadian Consensus Criteria [23]. None reported consuming immune-modulating drugs, while healthy controls were morbidity free, as diagnosed by participating medical doctors. The demographics of participants, including age, sex, BMI and the Short Form-36 Health Survey physical component summary [24] are reported in Table 1.

### 4.2. Biological Specimen Preparation

Peripheral blood was drawn from an antecubital vein into PAXgene Blood RNA tubes (Qiagen, Germantown, MD, USA) for the eight whole blood samples. For the virus amplification experiments, blood was collected into EDTA tubes, immediately placed on ice and transported to the laboratory where PBMCs were isolated via centrifugation using SepMate tubes (STEMCELL Technologies, Vancouver, BC, Canada), as previously described in ref. [15], and stored in liquid nitrogen. Extracellular vesicles were isolated from stored plasma via size exclusion chromatography using qEV10/35 nm columns (Izon Science, Christchurch, New Zealand) and concentrated with Amicon Ultra Centrifugal Filters (Millipore Sigma, Burlington, MA, USA).

### 4.3. In Vitro Co-Culture Experiments

#### 4.3.1. Cell Culture System

Experiments were conducted using a 24-well SUPER E-MIX^TM^ culture (Catalog #9202424 by Quidel—Diagnostic Hybrids, San Diego, CA, USA) containing two cell lines. One of them was genetically modified buffalo green monkey kidney cells (sBGMK), which are highly susceptible to enterovirus infection and provide a robust model for viral replication and cytopathic effect (CPE) detection. The second cell line was human lung carcinoma cells (A549), which are used to mimic human respiratory epithelium, which is relevant for studying enterovirus tropism and pathogenesis in a human-like environment. Upon arrival, cell cultures were incubated and monitored for morphology, with all procedures adhering to the manufacturer’s recommendations.

#### 4.3.2. Inoculation and Incubation

Once 70–80% confluence was reached, cultures were inoculated in triplicates either with PBMCs (from 4 to 6 mL), plasma-derived extracellular vesicles (10^6^ particles), or subject-matched PBMCs and extracellular vesicles. Plates were centrifuged at 700× *g* for 1 h at room temperature (20–25 °C), then incubated at 37 °C in a humidified 5% CO_2_ atmosphere using SUPER E-MIX^TM^ Refeed Medium (0.1% FBS, NEAA, 100 units/mL penicillin, and 100 µg/mL streptomycin).

#### 4.3.3. Monitoring, Passaging and Sampling

Cultures were monitored daily for cytopathic effects (CPE) via microscopy. Independent cultures inoculated with either PBMCs or extracellular vesicles were incubated for one week, blind passaged, and incubated for a second week, with sampling conducted at the end of each week. For experiments combining patient-matched PBMCs and extracellular vesicles, cultures were maintained for seven days, blind passaged to a new 24-well plate, and incubated for an additional seven days, with sampling performed at the end of the second week. Blind passaging was included to account for EVs that may not induce CPE until after passaging [25].

#### 4.3.4. Immunocytochemistry (ICC)

ICC targeting EV viral protein 1 (VP1) antigens was performed using the D3 IFA Enterovirus Kit (Diagnostic Hybrids, Athens, OH, USA), which employs FITC-conjugated EV VP1-specific monoclonal antibodies for comprehensive detection of human enteroviruses across species via immunofluorescence. Positive staining was verified using EV antigen control slides, following the manufacturer’s protocol.

### 4.4. RNA Extraction, Library Construction and Sequencing

Total RNA was extracted from PAXgene-stabilized whole blood using the PAXgene Blood miRNA Kit (PreAnalytiX, Hombrechtikon, Switzerland), according to the manufacturer’s instructions. RNA from PBMCs and in vitro co-culture experiments was extracted using the RNeasy Mini Kit (Qiagen, Valencia, CA, USA). Human heart total RNA (Takara Bio, Mountain View, CA, USA; PN: 636532) was used as background RNA for preparing the EV71 (ATCC^®^ VR-1775DQ™) dilution series. RNA quantity and integrity were assessed using the Qubit RNA high sensitivity quantitation assay (ThermoFisher, Waltham, MA, USA) and the Fragment Analyzer (Agilent, Santa Clara, CA, USA), respectively.

Library preparations were completed at Cornell’s Transcription Regulation and Expression Facility (TREx). In short, RNA was spiked with ERCC quality control RNA transcripts (Supplementary Table S1) to both monitor library construction and use as internal sensitivity standards in CATCH-EVseq experiments. Ribosomal RNA was removed by hybridization using the NEBNext rRNA Depletion Kit (New England Biolabs, Ipswich, MA, USA). TruSeq-barcoded RNAseq libraries were generated with the NEBNext Ultra II RNA Library Prep Kit (New England Biolabs, Ipswich, MA, USA). Each library was then quantified with a Qubit dsDNA High Sensitivity kit (ThermoFisher, Waltham, MA) and the size distribution was determined with a Fragment Analyzer (Agilent, Santa Clara, CA, USA) prior to pooling.

### 4.5. Probe Design

Probes were designed using unique human enteroviral genomes across EVA, EVB, EVC, and EVD species, downloaded on 7 March 2019, from both NCBI (National Center for Biotechnology Information) and BV-BRC (Bacterial and Viral Bioinformatics Resource Center, www.bv-brc.org). A probe set containing 49,847 probes was generated (Supplementary Table S1) using a total of 3410 genomes along with ten sequences from the ERCC (External RNA Controls Consortium). This common set of external RNA controls consists of a set of unlabeled, polyadenylated transcripts added to an RNA experiment to monitor library construction and provides an internal quantification standard based on variations in concentrations between each ERCC transcript provided (Thermo Fisher Scientific, ERCC RNA Spike-In Mix, Catalog #4456740, www.thermofisher.com).

The CATCH framework V1.2.0 [13] in Python V3.7 was used with the following constant parameters, including a probe length of 100 nt; a sliding along the input sequences, or probe stride, of 33 nt; the requirement for continuous nucleotides matches, or island of exact match, of 25 nt; the filtering out of polyA sequences longer than 20 nt to eliminate any potential polyA tails in downloaded EV genomes; and finally, the ‘probe expansion’ set to ‘ON’ meaning that ‘N’ nucleotides are converted to each of the four possible nucleotide substitutes ‘A, T, C, G’. Concerning the variable parameters, the number of allowed mismatches and size of cover extension are automatically chosen by CATCH based on the user chosen limit in probe design, which we limited to no more than 50,000 probes. The cover extension refers to the additional bases on either side of the target subsequence that a probe is considered to cover. Both variable parameters are justified because they are sequence dependent.

### 4.6. EV Sequences Enrichment and Sequencing

Individual cDNA libraries were pooled to a total size of 1.5 to 4 μg and processed according to the Twist Target Capture Enrichment Standard Hybridization Protocol [16]. Briefly, pooled cDNA libraries were dried using a vacuum concentrator (Savant SVC100 SpeedVac, Farmingdale, NY, USA). The CATCH designed EV-targeting probes synthesized by Twist were suspended in hybridization solution and then mixed with the reconstituted cDNA pool prepared in the presence of universal blockers. The hybridized cDNA targets were then bound to streptavidin beads via magnetic separation, put through several washes and then eluted directly prior to PCR amplification and subsequent purification. Each library was then validated and quantified using an Agilent Bioanalyzer High Sensitivity DNA Kit and a Qubit dsDNA High Sensitivity Quantitation Assay.

Library pools were then sequenced on either a MiSeq (2 × 250 bp), HiSeq (2 × 75 bp) or NovaSeq600 (2 × 150) platform. Total reads generated were reduced by eliminating any reads mapping to human (hg38) and *Chlorocebus sabaeus* (green monkey, chlSab2), due to the use of buffalo green monkey cells during in vitro cell culture experiments. The database of 3410 human EV reference genomes was indexed using Bowtie2. Unmapped reads were aligned to the EV genomes using the Bowtie2 read mapper. The location the of mapped reads was verified with Samtools (v1.13) and visualized using Interactive Genome Viewer (IGV, v2.19.7).

### 4.7. Analysis of Sequences

Parameters used for sequence data analysis:

- fastp -0.23.4: -z 4 -w 16 \ --length_required 50 --qualified_quality_phred 20 \ --trim_poly_g
- bowtie2-2.5.1: --very-sensitive
- STAR-2.7.11b
- HTSeq-2.0.9

## Figures and Tables

**Table 1:** Population characteristics.

		Controls	ME	*p*-Value
Sex	female	2	11	NA
	male	3	4	NA
Age	(mean of years )	50.4 ± 15.2	44 ± 11.7	0.4
BMI	(mean of kg·m^−2^)	29.2 ± 6.8	24.8 ± 8.2	0.3
SF-36—PCS	(median of scores)	51.3 & 59.9 *	23.4 ± 16.8	NA

The asterisk (*) denotes where data is provided for two of the five controls; the datapoints for the remaining three controls were not available, so no median was calculated. Note: The *p*-value for age and BMI were derived from a *t* test. No statistical analysis was conducted for SF-36 physical component summary (PCS) due to insufficient control data.

## Data Availability

The original contributions presented in this study are included in the article/Supplementary Materials. Further inquiries can be directed to the corresponding author.

## Supplementary Materials

The following supporting information can be downloaded at https://www.mdpi.com/article/10.3390/ijms27209065/s1.

## Abbreviations

The following abbreviations are used in this manuscript:

EV	enterovirus
ME	Myalgic Encephalomyelitis
ERCC	External RNA Controls Consortium
PBMC	peripheral blood mononuclear cells
ICC	immunocytochemistry
CPE	cytopathic effects

## References

[1] Centers for Disease Control and Prevention Non-Polio Enteroviruses—Outbreak & Surveillance. https://www.cdc.gov/non-polio-enterovirus/outbreak-surveillance/index.html.

[2] Bouin A., Gretteau P.A., Wehbe M., Renois F., N’Guyen Y., Lévêque N., Vu M.N., Tracy S., Chapman N.M., Bruneval P. (2019). Enterovirus Persistence in Cardiac Cells of Patients With Idiopathic Dilated Cardiomyopathy Is Linked to 5′ Terminal Genomic RNA-Deleted Viral Populations with Viral-Encoded Proteinase Activities. Circulation.

[3] Richardson S.J., Morgan N.G. (2018). Enteroviral infections in the pathogenesis of type 1 diabetes: New insights for therapeutic intervention. Curr. Opin. Pharmacol..

[4] Baj A., Colombo M., Headley J.L., McFarlane J.R., Liethof M.-A., Toniolo A. (2015). Post-poliomyelitis syndrome as a possible viral disease. Int. J. Infect. Dis..

[5] Helfferich J., Knoester M., Van Leer-Buter C.C., Neuteboom R.F., Meiners L.C., Niesters H.G., Brouwer O.F. (2019). Acute flaccid myelitis and enterovirus D68: Lessons from the past and present. Eur. J. Pediatr..

[6] Garzo-Caldas N., Ruiz-Sainz E., Vila-Bedmar S., Llamas-Velasco S., Hernández-Lain A., Ruiz-Morales J., Folgueira-López D., Villarejo-Galende A. (2017). Enteroviral T-cell encephalitis related to immunosuppressive therapy including rituximab. Neurology.

[7] Anderson S.M., Gold D., Olson G., Pisano J. (2022). Chronic aseptic meningitis caused by enterovirus in a humorally immunosuppressed adult patient presenting with sensorineural hearing loss: A case report. BMC Infect. Dis..

[8] Domingo E., Martin V., Perales C., Escarmis C. (2008). Coxsackieviruses and quasispecies theory: Evolution of enteroviruses. Curr. Top. Microbiol. Immunol..

[9] O’Neal A.J., Hanson M.R. (2021). The Enterovirus Theory of Disease Etiology in Myalgic Encephalomyelitis/Chronic Fatigue Syndrome: A Critical Review. Front. Med..

[10] Hanson M.R. (2023). The viral origin of myalgic encephalomyelitis/chronic fatigue syndrome. PLoS Pathog..

[11] Hyde B. (2020). Understanding Myalgic Encephalomyelitis.

[12] Gaudin M., Desnues C. (2018). Hybrid Capture-Based Next Generation Sequencing and Its Application to Human Infectious Diseases. Front. Microbiol..

[13] Metsky H.C., Siddle K.J., Gladden-Young A., Qu J., Yang D.K., Brehio P., Goldfarb A., Piantadosi A., Wohl S., Carter A. (2019). Capturing sequence diversity in metagenomes with comprehensive and scalable probe design. Nat. Biotechnol..

[14] Vidal D., Castro I.A., Lopez C.B. (2026). Implications of RNA virus persistence for post-acute sequelae and chronic inflammatory syndromes. Nat. Immunol..

[15] Nikolaidis M., Mimouli K., Kyriakopoulou Z., Tsimpidis M., Tsakogiannis D., Markoulatos P., Amoutzias G.D. (2019). Large-scale genomic analysis reveals recurrent patterns of intertypic recombination in human enteroviruses. Virology.

[16] Bioscience T. Twist Target Enrichment Standard Hybridization v1 Protocol. https://www.twistbioscience.com/resources/protocol/twist-target-enrichment-standard-hybridization-v1-protocol.

[17] Qiagen (2022). How Much DNA and RNA Can Be Expected from HUMAN blood Cells?.

[18] Briese T., Kapoor A., Mishra N., Jain K., Kumar A., Jabado O.J., Lipkin W.I. (2015). Virome Capture Sequencing Enables Sensitive Viral Diagnosis and Comprehensive Virome Analysis. mBio.

[19] World Health Organization, Regional Office for Europe (2015). Enterovirus Surveillance Guidelines: Guidelines for Enterovirus Surveillance in Support of the Polio Eradication Initiative.

[20] World Health Organization (2004). Polio Laboratory Manual.

[21] Benschop K.S.M., Zwagemaker F., Andersson-Li L., Andres C., Anton A., Berengua C., Berginc N., Bessaud M., Bisseux M., Bujaki E. (2026). Next generation sequencing approaches for the detection and characterization of enteroviruses in clinical, public health, and research settings: Expert view of the European non-polio enterovirus network (ENPEN). J. Clin. Virol..

[22] Genoni A., Canducci F., Rossi A., Broccolo F., Chumakov K., Bono G., Salerno-Uriarte J., Salvatoni A., Pugliese A., Toniolo A. (2017). Revealing enterovirus infection in chronic human disorders: An integrated diagnostic approach. Sci. Rep..

[23] Carruthers B.M., Jain A.K., De Meirleir K.L., Peterson D.L., Klimas N.G., Lerner A.M., Bested A.C., Flor-Henry P., Joshi P., Powles A.C.P. (2003). Myalgic Encephalomyelitis/Chronic Fatigue Syndrome. J. Chronic Fatigue Syndr..

[24] Ware J.E., Sherbourne C.D. (1992). The MOS 36-item short-form health survey (SF-36). I. Conceptual framework and item selection. Med. Care.

[25] Smura T., Natri O., Ylipaasto P., Hellman M., Al-Hello H., Piemonti L., Roivainen M. (2015). Enterovirus strain and type-specific differences in growth kinetics and virus-induced cell destruction in human pancreatic duct epithelial HPDE cells. Virus Res..

